# Postpartum Oxytocin Treatment via the Mother Reprograms Long-Term Behavioral Disorders Induced by Early Life Stress on the Plasma and Brain Metabolome in the Rat

**DOI:** 10.3390/ijms25053014

**Published:** 2024-03-05

**Authors:** Sara Morley-Fletcher, Alessandra Gaetano, Vance Gao, Eleonora Gatta, Gilles Van Camp, Hammou Bouwalerh, Pierre Thomas, Ferdinando Nicoletti, Stefania Maccari

**Affiliations:** 1 Unité de Glycobiologie Structurale et Fonctionnelle, GlycoStress Team, CNRS, UMR 8576, UGSF, Université de Lille, F-59000 Lille, France; sara.morley-fletcher@univ-lille.fr (S.M.-F.); alessandra.gaetano@univ-lille.fr (A.G.); vance.gao@univ-lille.fr (V.G.); eleonora.gatta@northwestern.edu (E.G.); gilles.van-camp@univ-lille.fr (G.V.C.); hammou.bouwalerh@univ-lille.fr (H.B.); 2 INSERM (U-1172) Laboratoire Lille Neuroscience & Cognition, équipe Plasticity & Subjectivity, Plateforme CURE, Hôpital Fontan, CHU de Lille, Psychiatry Department, Université de Lille, F-59000 Lille, France; pierre.thomas@univ-lille.fr; 3 Department of Physiology and Pharmacology “V. Erspamer”, University Sapienza of Rome, 00185 Roma, Italy; ferdinandonicoletti@hotmail.com; 4 IRCCS Neuromed, 86077 Pozzilli, Italy; 5 Department of Science and Medical-Surgical Biotechnology, University Sapienza of Rome, 00185 Roma, Italy

**Keywords:** early life stress, plasma, prefrontal cortex, oxytocin, secondary bile acid, glutathione, behavior, corticosterone, glucose

## Abstract

The rat model of perinatal stress (PRS), in which exposure of pregnant dams to restraint stress reduces maternal behavior, is characterized by a metabolic profile that is reminiscent of the “metabolic syndrome”. We aimed to identify plasma metabolomic signatures linked to long-term programming induced by PRS in aged male rats. This study was conducted in the plasma and frontal cortex. We also investigated the reversal effect of postpartum carbetocin (Cbt) on these signatures, along with its impact on deficits in cognitive, social, and exploratory behavior. We found that PRS induced long-lasting changes in biomarkers of secondary bile acid metabolism in the plasma and glutathione metabolism in the frontal cortex. Cbt treatment demonstrated disease-dependent effects by reversing the metabolite alterations. The metabolomic signatures of PRS were associated with long-term cognitive and emotional alterations alongside endocrinological disturbances. Our findings represent the first evidence of how early life stress may alter the metabolomic profile in aged individuals, thereby increasing vulnerability to CNS disorders. This raises the intriguing prospect that the pharmacological activation of oxytocin receptors soon after delivery through the mother may rectify these alterations.

## 1. Introduction

The Developmental Origins of Adult Health and Disease (DOHaD) theory postulates that adverse environmental exposures during critical in-utero and early postnatal stages can permanently alter physiological responses, leading to functional impairments and adult disorders [1]. Furthermore, it has been suggested that mechanisms involved in fetal metabolic programming could lead to the discovery of placental biomarkers at birth that predict later-life metabolic risk [2]. These factors not only determine the risk for adult-onset disorders but also influence the aging process and longevity [3,4,5,6].

This process, known as developmental programming, has a lifelong impact on stress response, emotional behavior, metabolism, and cerebral plasticity [7,8]. Crucial factors influencing this programming process include developmental malnutrition, insufficient parental care, stressors, and hypoxia [9,10,11]. In particular, maternal behavior programming, a complex process where maternal experiences during pregnancy are instrumental in determining maternal care and influencing offspring’s long-term health, has gained attention in neuroscience and biology, highlighting the crucial role of maternal care in an individual’s lifelong well-being and responses to environmental challenges.

The rat model of perinatal stress (PRS), in which exposure of pregnant dams to restraint stress reduces maternal behavior [12], has face, construct, and pharmacological validity as an epigenetic model of stress-related disorders [8], where the age-dependent threshold for neurochemical and behavioral dysfunction is lowered [13]. The PRS rat model exhibits in the offspring stress-related hallmarks, including a lifelong disruption in the activity and feedback regulation of the hypothalamic–pituitary–adrenal stress axis [8], disrupted circadian rhythms and sleep-wake cycle, and a systemic proinflammatory profile in both adults [8] and aged rats [14]. During aging, PRS rats are also characterized by a display of hyperglycemia both under fasting conditions and in response to a glucose load, whereas insulinemia is not affected in adult and old PRS male rats and middle-aged (10-month-old) PRS females [15].

The identification of specific metabolites in the plasma holds promise as potential biomarkers for stress-related disorders. Altered levels of specific metabolites may signal the presence, severity, or progression of these disorders, aiding in diagnosis and prognosis. The metabolome, encompassing all small molecules characterizing a biological system, undergoes alterations with age, reflecting age-related changes in physiological function [16,17,18,19]. Many psychiatric and somatic diseases disrupt metabolism, leading to enduring metabolic signatures for a particular disease [20]. Neurodegenerative diseases, which are more prevalent in older populations, often have links to inflammation and metabolic dysregulation [21,22,23]. The impact of circulating metabolites on neurological development and preservation is particularly relevant to aging. Notably, as individuals age, there are significant changes in the composition and function of the gut microbiota, commonly termed “gut dysbiosis”. Metabolic signatures associated with stress in the plasma may offer early indicators of health risks in later life and introduce novel candidates for peripheral biomarker detection with diagnostic value. Additionally, the ability of specific metabolites to cross the blood-brain barrier highlights their potential impact on cognitive function and neurological health in aging individuals.

Oxytocin (OT), a neurohypothalamic hormone crucial for maternal bonding and social attachment, exerts anti-stress effects and positively regulates glucose homeostasis, central and systemic immune activity, and microbiota composition. The OT system is also involved in various aspects of social cognition and prosocial behavior. Specifically, OT has been examined in the context of social memory, emotion recognition, cooperation, trust, empathy, and bonding. For example, while evidence is somewhat mixed, intranasal OT appears to benefit aspects of socioemotional functioning [24]. We have shown that reduced maternal behavior caused by gestational stress was corrected through activation of the OT system, i.e., post-partum treatment with the OT agonist, carbetocin (Cbt). Targeting the OT system in the stressed mother has the potential to reverse the PRS phenotype at the behavioral and molecular levels in both adult and aged offspring [12]. Therefore, the anti-stress action of the OT system, combined with its effects on glucose and lipid metabolism, makes it a promising target for the treatment of stress-related disorders. In this study, we aimed to identify plasma metabolomic signatures linked to long-term programming induced by perinatal stress in aged animals. We also investigated the reversal effect of postpartum Cbt on these signatures, along with its impact on deficits in cognitive, social, and exploratory behavior associated with aging. Global metabolic profiling was conducted on plasma obtained from the aged offspring of stressed and unstressed mothers, both treated and untreated with Cbt, as well as in the prefrontal cortex.

## 2. Results

### 2.1. Experimental Design

**Figure 1:**
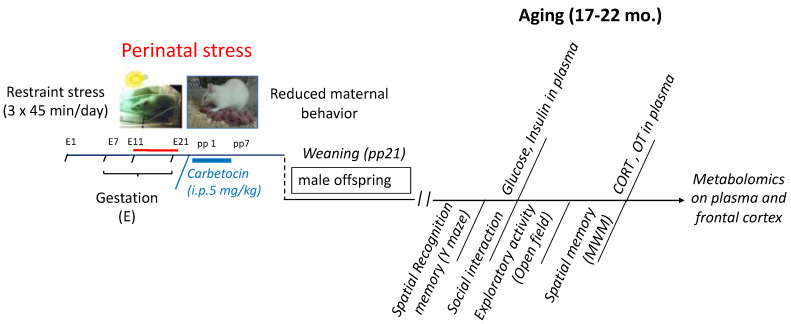
Experimental timeline. Restraint stress was performed during the last 10 days of gestation. Dams were treated with carbetocin i.p. during the first seven days of lactation. This experiment included 4 different experimental groups for the progeny: the PRS group whose mothers were treated with carbetocin (PRS-Cbt) or vehicle (PRS-Veh) and the corresponding control unstressed groups (Cont-Cbt or Cont-Veh). Behavioral tests were performed during aging (17–22 mo). Tissues for endocrinological and metabolomic analyses were collected when animals were 22 months of age.

As illustrated in Figure 1, male offspring aged 17–22 months, born to either stressed or unstressed control mothers, which were administered either vehicle or carbetocin were used. The resulting offspring were assigned to four experimental groups for the study: PRS-Veh, PRS-Cbt, and their respective control counterparts (i.e., unstressed and vehicle-treated). Behavioral assessments were conducted between 9:00 a.m. and 1:00 p.m. Brain tissue samples were collected at 22 months of age.

### 2.2. Behavioral Analysis

We carried out a behavioral characterization of aged progeny (17 to 22 months) of stressed and unstressed dams treated with carbetocin and vehicle. The aged offspring were tested for discrimination and spatial recognition memory, social interaction, spatial learning, and exploratory activity in the Y-maze, social interaction test, Morris water maze, and open field, respectively. The time interval between performing the various tests was 1 to 2 months to avoid behavioral test interference.

**Figure 2:**
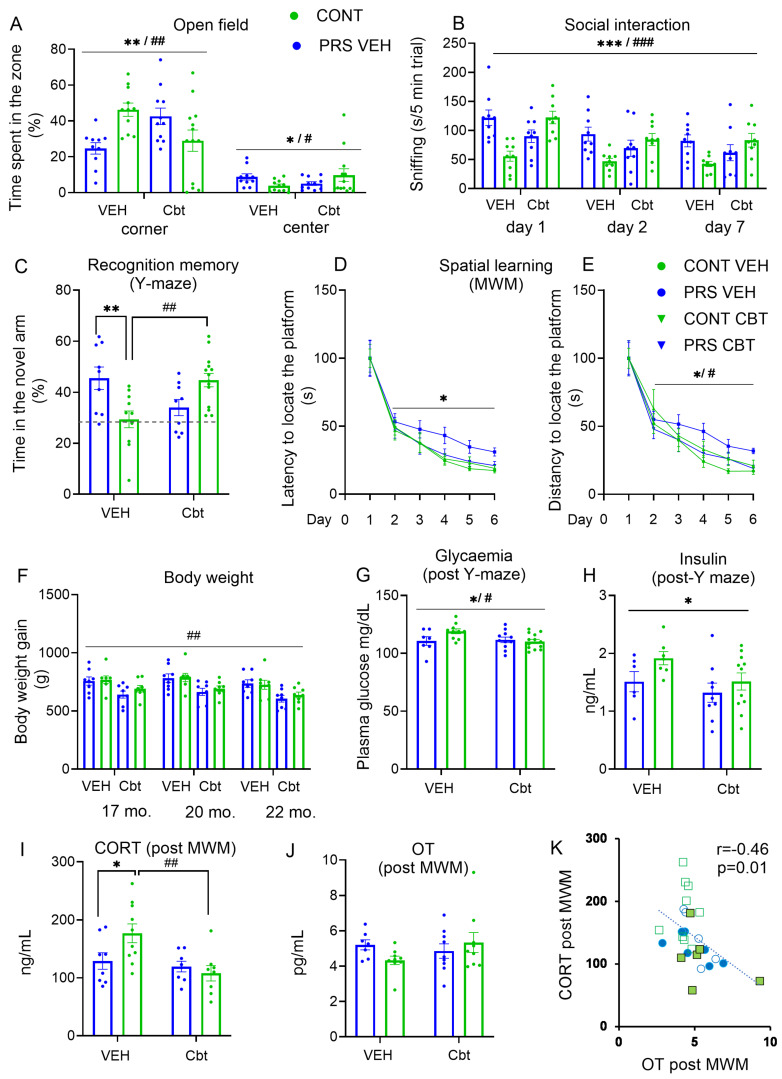
Long-term alterations induced by PRS on behavior and metabolism and reversal by carbetocin. (**A**) Exploratory activity in the open field (n = 10–13 rats/group) represented as the time spent (%) in the corners and the center. (**B**) Social interaction (n = 9 rats/group) represented by sniffing activity (sec/5 min trial). (**C**) Spatial recognition memory in the Y-maze (n = 9–14 rats/group); the time spent (%) was the parameter analyzed. (**D**,**E**) Spatial learning on the Morris water maze (MWM) (n = 9–13 rats/group); the latency and distance to find the hidden platform, respectively. (**F**) Weight of the aged progeny at 17, 20, and 22 months (n = 8 rats/group). (**G**–**J**) Plasma levels of glucose (mg/dL) (n = 7–13 rats/group), insulin (ng/mL) (n = 6–11 rats/group), corticosterone (ng/mL) (n = 8–10 rats/group), and oxytocin (pg/mL) (n = 7–10 rats/group), respectively. (**K**) Pearson correlation between corticosterone and oxytocin levels (n = 6 rats/group). Values are expressed as means ± S.E.M. * indicates the PRS effect and # indicates the Cbt effect. Specifically, in the figure, PRS × Cbt interaction */# *p* < 0.05; **/## *p* < 0.01; ***/### *p* < 0.001. PRS main effect * *p* < 0.05; ** *p* < 0.01; *** *p* < 0.001; Cbt main effect # *p* < 0.05; ## *p* < 0.01; ### *p* < 0.001.

The exploratory activity was assessed in the open field (Figure 2A). PRS offspring of vehicle-treated mothers showed an increased time spent in the periphery and corners of the open field and a reduction in time spent in the center of the apparatus. Administration of Cbt to lactating mothers reversed the exploratory behavior observed in PRS animals in each of the three analyzed zones of the open field (periphery: PRS × Cbt treatment interaction, F_(1,41)_ = 5.48, *p* < 0.05, n = 10–13 rats/group; corner: PRS × Cbt interaction, F_(1,41)_ = 13.73, *p* < 0.01, n = 10–13 rats/group, Neuman–Keuls post hoc test: ** *p* < 0.01 PRS-vehicle vs. Cont-Veh and # *p* < 0.05 PRS-Cbt vs. PRS-vehicle; center: PRS × Cbt interaction, F_(1,41)_ = 4.44, *p* < 0.05, n = 10–13 rats/group). In the social interaction test (Figure 2B), at days 1, 2, and 7, the aged PRS offspring of vehicle-treated mothers showed reduced sniffing during social interaction toward a juvenile as compared to the respective control animals. Postpartum carbetocin treatment to dams abolished the differences between control and PRS rats in all days of testing (PRS × Cbt interaction, F_(1,32)_ = 16.51, *p* < 0.001; days main effect, F_(2,64)_ = 19.365, *p* < 0.001, n = 9 rats/group/testing day).

The study of spatial recognition memory (Figure 2C) showed that PRS impaired memory performance in the aged offspring of vehicle-treated mothers, with a significative reduction of the time spent in the novel arm of Y-maze (below the 33% cut-off chance level of random exploration) after an inter-trial interval (ITI) of 6 h. Cbt administration to lactating dams abolished the differences between control and PRS rats (*PRS × Cbt interaction*, F_(1,39)_ = 16.03, *p* < 0.01, n = 9–14 rats/group; Neuman–Keuls post hoc test: ** *p* < 0.01 PRS-vehicle vs. Cont-vehicle and ## *p* < 0.01 PRS-Cbt vs. PRS-vehicle). After an ITI of 24 h, spatial recognition memory fell in every group, with no difference being observed among groups.

Spatial learning (Figure 2C,D) assessment indicated that PRS impaired learning tasks in the aged progeny of vehicle-treated mothers, with a significantly increased latency to find the hidden platform at days 4, 5, and 6 (Figure 2C); conversely, carbetocin to lactating dams abolished the differences between control and PRS rats in all days of testing (PRS main effect, F_(1,40)_ = 5.33, *p* < 0.05; trend for PRS × Cbt effect, F_(1,40)_ = 3.49, *p* = 0.06, n = 9–13 rats/group/testing day). The same profile was found for the distance to reach the platform (Figure 2D: PRS × Cbt interaction, F_(1,40)_ = 5.51, *p* < 0.05; days main effect, F_(5,200)_ = 50.26, *p* < 0.01, n = 9–13 rats/group/testing day).

### 2.3. Physiological Analysis

Carbetocin reduces body weight gain in the aged offspring and prevents changes in fasting plasma glucose, corticosterone, and oxytocin levels induced by gestational stress.

The body weight of aged rats (Figure 2F) was not changed by PRS. However, we observed that aged offspring of stressed and unstressed mothers treated with carbetocin showed a reduction in body weight at 17 months, which persisted until 22 months (PRS × Cbt interaction F_(1,39)_ = 16.03, *p* < 0.01, n = 9–14 rats/group).

Glucose and insulin levels were measured after the assessment of spatial recognition memory in aged rats from stressed and unstressed mothers treated with carbetocin or vehicle (Figure 2G,H). We observed that elderly PRS rats reared from vehicle-treated mothers showed a significant increase in blood glucose, which was restored to normal levels with carbetocin treatment (Figure 2G: Cbt main effect, F_(1,28)_ = 11.49, *p* < 0.01, n = 8 rats/group). A similar profile was observed for insulin, while we observed a trend for the Cbt effect (Figure 2H: PRS main effect, F_(1,29)_ = 3.48, *p* < 0.01; Cbt main effect, F_(1,29)_ = 3.43, *p* = 0.07, n = 6–11 rats/group). We measured corticosterone and oxytocin levels in peripheral blood collected after the last trial in the Morris water maze (a stressful condition) and showed that the HPA axis was hyper-reactive in aged PRS offspring, as well as a phenotype that was reversed by administration of carbetocin to lactating mothers (Figure 2I: PRS × Cbt interaction, F_(1,30)_ = 4.46, *p* < 0.05, Neuman–Keuls post hoc test: * *p* < 0.05 PRS-vehicle vs. Cont-vehicle and ## *p* < 0.01 PRS-Cbt vs. PRS-vehicle n = 8–10 rats/group). No changes in plasma oxytocin levels were observed (Figure 2J), while a strong negative correlation was found between corticosterone and oxytocin levels, which highlights the lifelong impairment of the stress/anti-stress balance induced by PRS (Figure 2K, r = −0.46, *p* = 0.01).

Maternal behavior and Pearson’s correlations (Figure S1). The analysis of active maternal behavior (nursing behavior, grooming, licking, carrying pups) was performed in stressed and unstressed dams receiving either vehicle or carbetocin during the first week postpartum (Figure S1A). As previously demonstrated [12], gestational stress impaired maternal behavior, which was calculated as the mean of 3 days. Carbetocin administration to lactating dams abolished the differences between unstressed and stressed mothers (PRS *×* Cbt interaction: F_(1,43)_ = 10.32, *p* < 0.01, n = 8–15 rats/group; Neuman–Keuls post hoc test: * *p* < 0.05 stressed dams-vehicle vs. unstressed dams-vehicle and ### *p* < 0.001 stressed dams-Cbt vs. stressed dams-vehicle). Partial correlations (Figure S1B–F) were performed to examine the significant relationship between maternal behavior and the following behavioral and endocrinological parameters: (i) peripheral markers (corticosterone, glucose, and insulin); (ii) spatial recognition memory (Y-maze) and social interaction tests. Spatial memory performance in the Y-maze (6-h ITI; Figure S1B) and social interaction (sum of sniffing activity on test days 1, 2, and 7; Figure S1C) of aged offspring of stressed and unstressed dams receiving either vehicle or carbetocin were positively correlated with maternal behavior (r = 0.32 *p* < 0.05; r = 0.44, *p* < 0.01, respectively). Conversely, corticosterone (Figure S1D), glucose (Figure S1E), and insulin (Figure S1F) were negatively correlated with maternal behavior (r = −0.47 *p* < 0.01; r = −0.32, *p* < 0.05; r = −0.43, *p* < 0.01, respectively).

### 2.4. Untargeted Metabolomic Analysis

**Figure 3:**
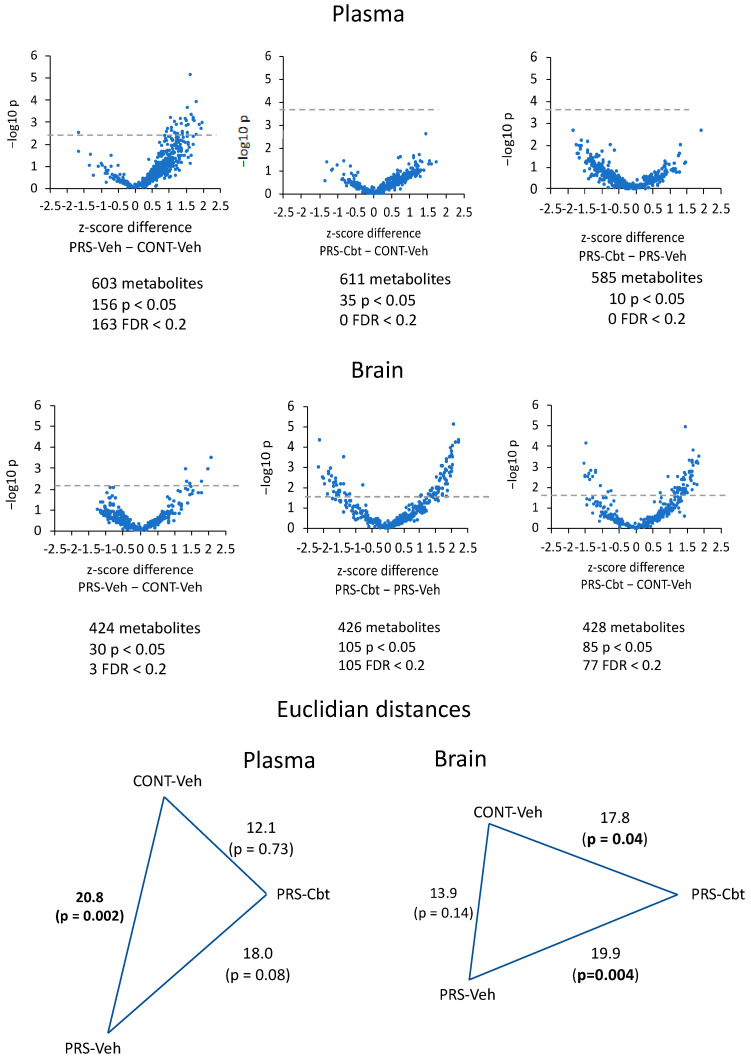
Metabolomic effect-size. Volcano plot and Euclidian distances of the plasma and brain metabolome. The dashed lines in the volcano plots represent the threshold of false discovery rate < 0.2. The significance of the Euclidean distance between groups was calculated by PERMANOVA. (n = 6 rat/group).

We visualized the changes in metabolite expression by creating Volcano plots to represent the direction, magnitude, and significance of these alterations (Figure 3). In the plasma, out of the 603 analyzed metabolites, 156 were significantly increased in vehicle-treated PRS compared to vehicle-treated Cont. Cbt treatment effectively reduced the expression of these metabolites in the PRS group, leaving only 35 metabolites that remained significantly different in Cbt-treated PRS rats compared to the Veh-treated PRS. When comparing PRS-Cbt rats with the Cont-vehicle group, among 585 metabolites, only 10 remained significantly different, indicating a correction by Cbt of about 98% with respect to the PRS group. This reduction is visually represented by the halving of the Euclidean distance between Cbt-treated PRS rats and vehicle-treated control unstressed rats, in comparison to the distance between PRS and control vehicle-treated groups or vehicle- and Cbt-treated PRS rats. In contrast, the Volcano plot representation for the brain showed that 30 out of 424 metabolites were differentially expressed between vehicle-treated PRS and unstressed rats. Cbt in the PRS group differentiated a total of 105 metabolites out of the 426 measured. When comparing Cbt-treated PRS rats with the unstressed group treated with vehicle, 85 metabolites remained different out of the 428 considered, indicating a correction by Cbt of about 80% between the two groups. The Euclidean distance indicated an opposite profile in the brain vs. plasma. The distance between vehicle-treated PRS and unstressed groups or vehicle- and Cbt-treated PRS groups remained significantly higher compared to the distance between PRS and control rats of the vehicle-treated groups.

Metabolomics and sub-pathway analysis. Several sub-pathways were identified as being modified by PRS, Cbt, or both.

**Figure 4:**
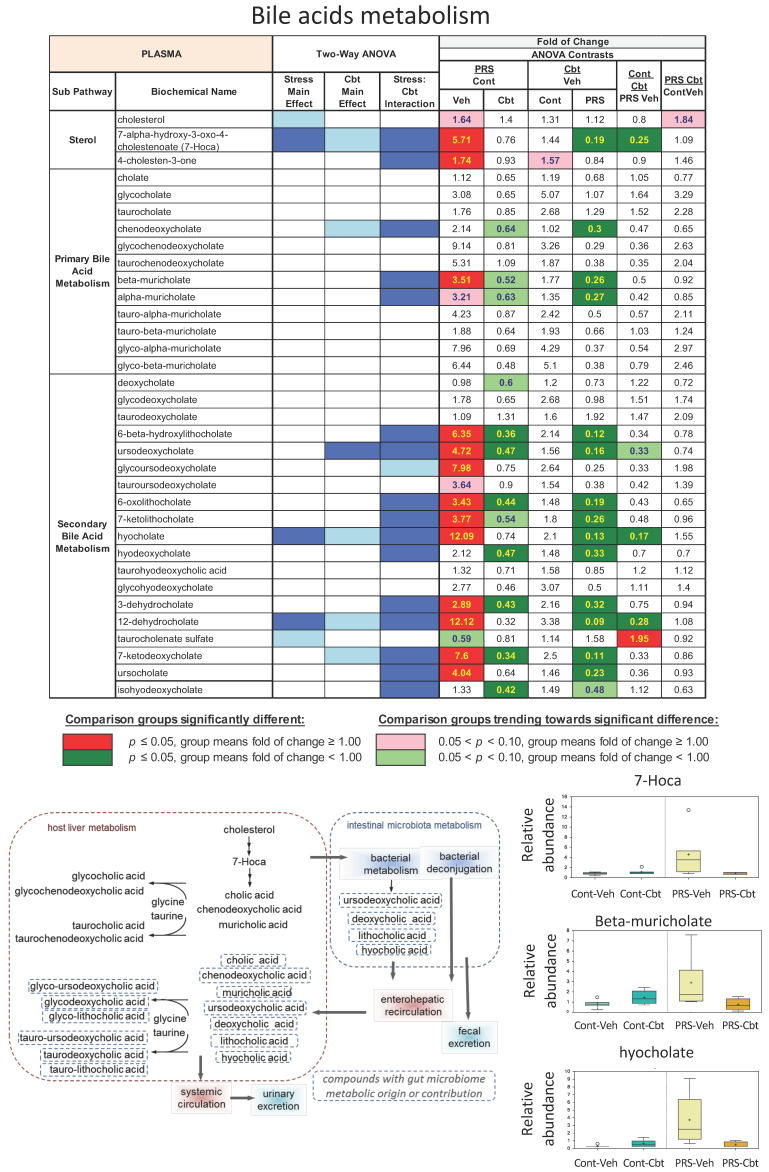
Long-term impact of PRS and carbetocin on bile acid metabolism in plasma. PRS elevated metabolites of bile acids’ specifically secondary acid pathways, and Carbetocin restored it to control unstressed levels. This table illustrates two-way ANOVA and ANOVA ratio contrasts for intergroup comparisons. Fold change values between groups are shown. In the two-way ANOVA, blue cells indicate a significant ANOVA effect *p* < 0.05, and the light blue indicates that *p* narrowly missed the statistical cutoff for ANOVA significance, 0.05 < *p* < 0.10. In the ANOVA ratio contrast, red cells indicate a significant difference (*p* < 0.05) between groups, with a metabolite ratio > 1.00 (upregulation), while green cells indicate a significant difference for metabolite ratios < 1.00 (downregulation). Box plots display the median value, upper quartile limit, and lower quartile limit. The solid bar across the box represents the median value of those measured, while + represents the mean. The following metabolites are included: 7-Hoca, beta-muricholate, and hyocholate (n = 6 rat/group).

In the plasma, the statistical summary analysis of data (Metabolon Inc., Germany, Head Quarters Morrisville, NC, USA) revealed the most significant inter-group metabolomic difference between groups. We observed a noticeable effect of PRS on bile acid (BA) metabolism (Figure 4). Specifically, among vehicle-treated PRS rats compared to vehicle-treated unstressed controls, all primary BAs and precursors were increased, with cholesterol, 7-Hoca, and the primary BA beta-muricholate and alpha-muricholate reaching trending or significant cutoffs. Treatment with Cbt decreased these compounds, particularly 7-Hoca, and their levels did not differ among Cbt-treated PRS rats and vehicle-treated unstressed rats. Furthermore, several secondary BAs, including the muricholate product, hyocholate (a.k.a., gamma-muricholate), behaved similarly, i.e., increasing with PRS and decreasing to control levels with Cbt treatment. As muricholate is a major BA in rodents, these results suggest that PRS programs a life-long increase in BA synthesis.

**Figure 5:**
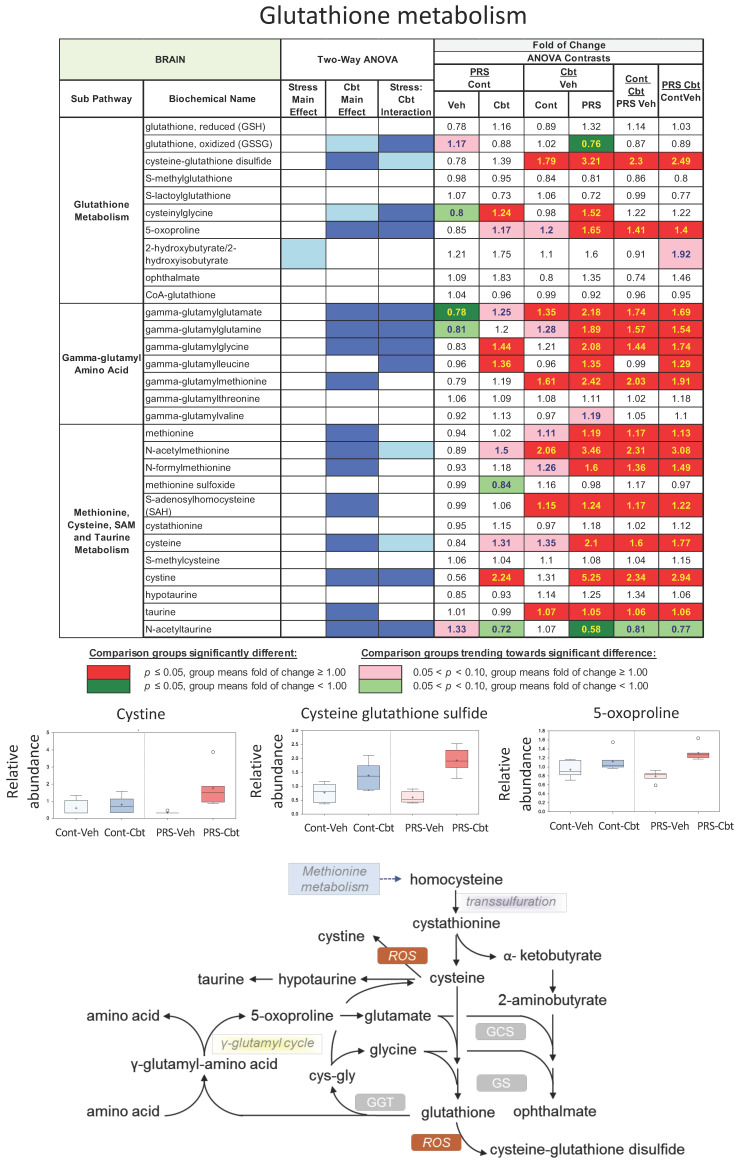
Long-term impact of PRS and carbetocin on glutathione metabolism in the brain prefrontal cortex. This table illustrates two-way ANOVA and ANOVA ratio contrasts for intergroup comparisons. Fold change values between groups are shown. In the two-way ANOVA, blue cells indicate a significant ANOVA effect *p* < 0.05, and the light blue indicates that *p* narrowly missed the statistical cutoff for ANOVA significance, 0.05 < *p* < 0.10. In the ANOVA ratio contrast, red cells indicate a significant difference (*p* < 0.05) between groups, with a metabolite ratio > 1.00 (upregulation), while green cells indicate a significant difference for metabolite ratios < 1.00 (downregulation). Box plots display the median value, upper quartile limit, and lower quartile limit. The solid bar across the box represents the median value of those measured, while + represents the mean. The following metabolites are included: cystine, cysteine-glutathione disulfide, and 5-oxoproline (n = 6 rat/group).

In the brain, the statistical summary analysis of data (Metabolon Inc., Germany, Head Quarters USA) revealed that PRS only had a marginal effect on the brain metabolome. PRS and Cbt caused alterations in brain glutathione metabolism (Figure 5), with Cbt inducing more pronounced effects. Cysteine and γ-glutamylcysteine synthase (GCS) are the rate-limiting substrate and enzyme, respectively, in glutathione synthesis. Cbt treatment caused a trend of an increase in brain cysteine levels in unstressed rats, and it had a significant increase in PRS rats, although it failed to induce changes in the cysteine precursor cystathionine. A similar pattern emerged in biochemical markers of glutathione turnover, such as 5-oxoproline and a subset of gamma-glutamyl amino acids, which showed a trend to an increase in response to Cbt in unstressed rats and were significantly increased by Cbt in PRS rats.

**Figure 6:**
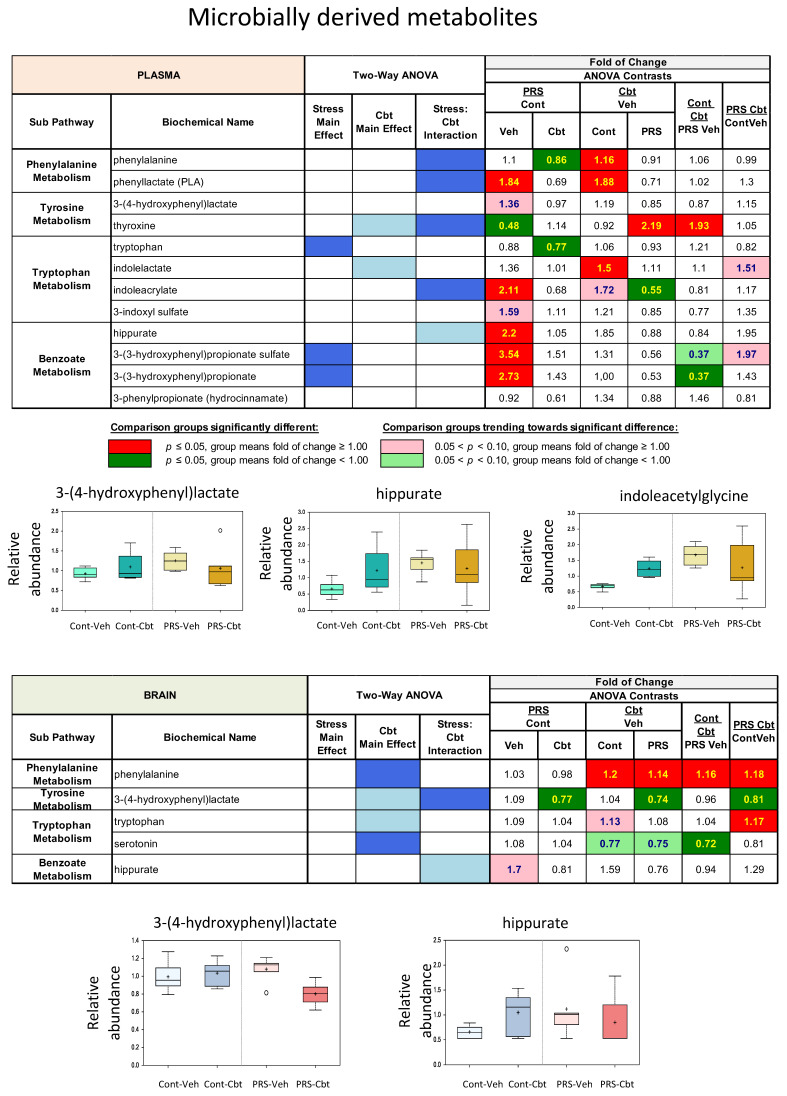
Long-term impact of PRS and carbetocin on microbially derived sub-pathways in plasma and brain prefrontal cortex. This table illustrates two-way ANOVA and ANOVA ratio contrasts for intergroup comparisons. Fold change values between groups are shown. In the two-way ANOVA, blue cells indicate a significant ANOVA effect *p* < 0.05, and the light blue indicates that *p* narrowly missed the statistical cutoff for ANOVA significance, 0.05 < *p* < 0.10. In the ANOVA ratio contrast, red cells indicate a significant difference (*p* < 0.05) between groups, with a metabolite ratio > 1.00 (upregulation), while green cells indicate a significant difference for metabolite ratios < 1.00 (downregulation). Box plots display the median value, upper quartile limit, and lower quartile limit. The solid bar across the box represents the median value of those measured, while + represents the mean. The following metabolites are included: 3-(4-hydroxyphenyl)lactate, Hippurate, and indoleacetylglycine (n = 6 rat/group).

**Figure 7:**
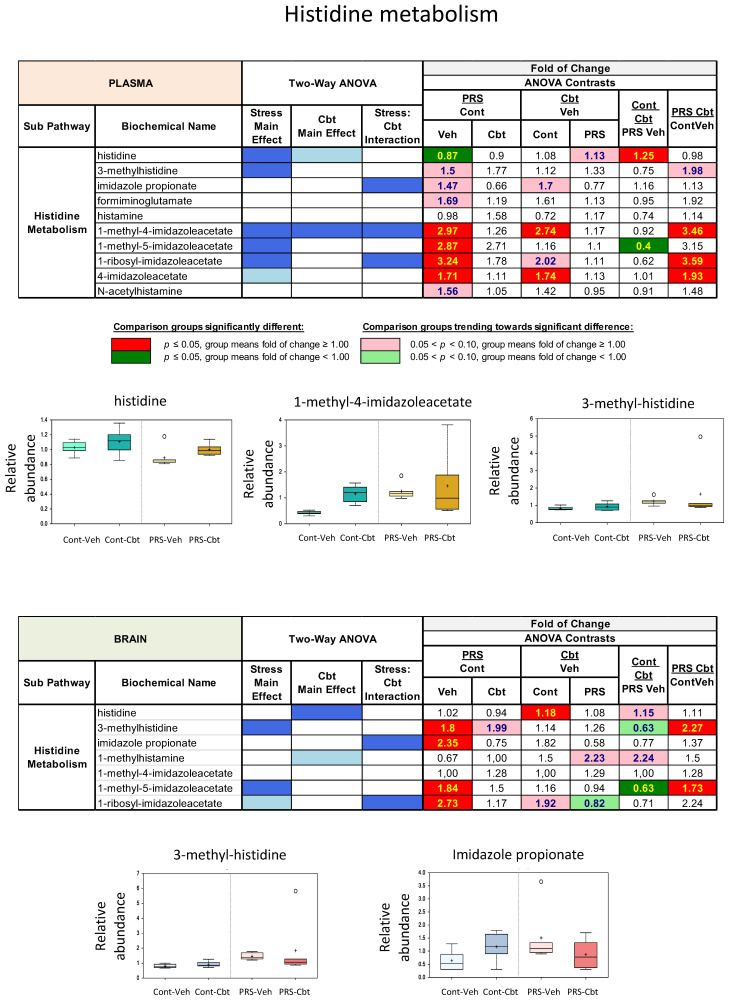
Long-term impact of PRS and carbetocin on histidine (inflammation) derived sub-pathways in plasma and brain prefrontal cortex. This table illustrates two-way ANOVA and ANOVA ratio contrasts for intergroup comparisons. Fold change values between groups are shown. In the two-way ANOVA, blue cells indicate a significant ANOVA effect *p* < 0.05, and the light blue indicates that *p* narrowly missed the statistical cutoff for ANOVA significance, 0.05 < *p* < 0.10. In the ANOVA ratio contrast, red cells indicate a significant difference (*p* < 0.05) between groups, with a metabolite ratio > 1.00 (upregulation), while green cells indicate a significant difference for metabolite ratios < 1.00 (downregulation). Box plots display the median value, upper quartile limit, and lower quartile limit. The solid bar across the box represents the median value of those measured, while + represents the mean. The following metabolites are included: histidine, 1-methyl4-imidazoleacetate, 3-methyl-histidine, and imidazole propionate (n = 6 rat/group).

**Figure 8:**
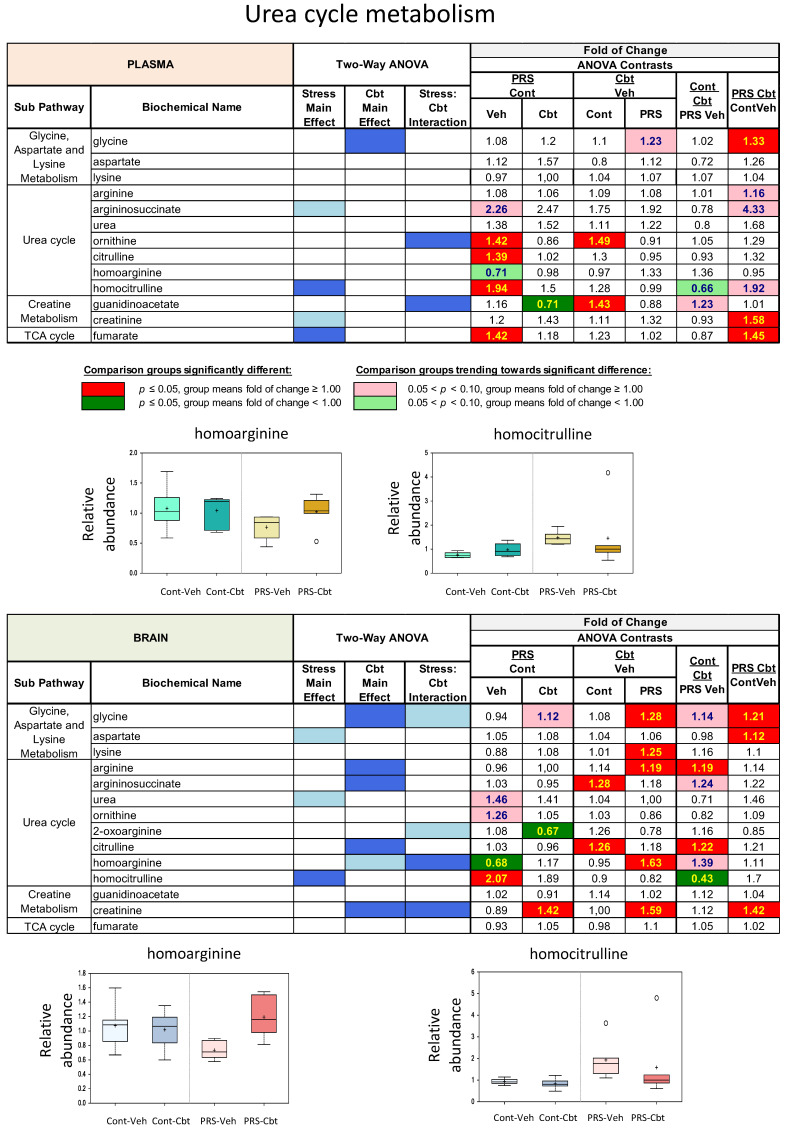
Long-term impact of PRS and carbetocin on a urea cycle sub-pathway in the plasma and brain prefrontal cortex. This table illustrates two-way ANOVA and ANOVA ratio contrasts for intergroup comparisons. Fold change values between groups are shown. In the two-way ANOVA, blue cells indicate a significant ANOVA effect *p* < 0.05, and the light blue indicates that *p* narrowly missed the statistical cutoff for ANOVA significance, 0.05 < *p* < 0.10. In the ANOVA ratio contrast, red cells indicate a significant difference (*p* < 0.05) between groups, with a metabolite ratio > 1.00 (upregulation), while green cells indicate a significant difference for metabolite ratios < 1.00 (downregulation). Box plots display the median value, upper quartile limit, and lower quartile limit. The solid bar across the box represents the median value of those measured, while + represents the mean. The following metabolites are included: homoarginine and homocitrulline (n = 6 rat/group).

Shared metabolomic pathways between plasma and brain metabolome. We identified three major sub-pathways with PRS and Cbt effects being shared between plasma and brain (Figure 6, Figure 7 and Figure 8) such as microbially derived metabolism, histidine, and urea cycle. Indeed, microbially derived compounds such as the metabolites of phenylalanine, tryptophane, and benzoate were enhanced by PRS in plasma (Figure 6). In particular, we observed an increase in the phenylalanine derivative, phenyl-lactate (PLA), the indole metabolites of tryptophan, indoleacrylate, and indoleacetylglycine, and the benzoate metabolites, hippurate, 3-(3-hydroxyphenyl) propionate, and 3-(3-hydroxyphenyl) propionate sulfate. In unstressed rats, Cbt treatment caused a trend to an increase in these metabolites, whereas in PRS rats Cbt treatment reduced these compounds, particularly the indol derivatives, approximately to the same levels observed in vehicle-treated unstressed rats. In the brain, Cbt treatment significantly reduced the increased 3-(4-hydroxyphenyl) lactate levels in the PRS group, and levels did not differ between Cbt-treated PRS rats and vehicle-treated unstressed rats.

Histidine metabolism was also altered by PRS (Figure 7) in the plasma and brain, with PRS significantly decreasing histidine levels compared to vehicle-treated unstressed rats and Cbt reversing these changes. Specifically, PRS significantly reduced histidine levels compared to unstressed controls, and Cbt treatment reversed changes in histidine levels caused by PRS. PRS also caused increases in plasma 3-methylhistidine levels, which, in this case, were further increased by Cbt treatment. Imidazole propionate is a bacterially derived catabolite of trans-urocanate, and PRS increased its levels in the plasma, indirectly suggesting that PRS causes long-lasting changes in the metabolic activity of the microbiome. Cbt increased levels of microbially produced metabolites in unstressed rats but decreased their levels in PRS rats. While plasma histamine levels were not significantly different among the experimental groups, several of its catabolic products, such as 1-methyl-5-imidazoleacetate, 1-ribosyl-imidazoleacetate, 1-methyl-4-imidazoleacetate, and 4-imidazoleacetate, were significantly elevated in the plasma of vehicle-treated PRS rats compared to vehicle-treated unstressed rats, which is indicative of stimulated histamine turnover. Cbt treatment further increased these levels in the plasma of unstressed rats, with the exception of 1-methylhistamine, which showed a trend to increase in PRS rats treated with Cbt.

Finally, PRS caused major changes in ureal cycle metabolites (Figure 8). In vehicle-treated rats, the PRS group showed trending and significant reductions in plasma homoarginine levels, as well as significant elevations in plasma homocitrulline levels. PRS also increased the levels of ornithine and citrulline, which is indicative of a dysfunctional urea cycle. Cbt treatment had a marginal effect on plasma arginine metabolites, although it reversed the effect of PRS on homoarginine, ornithine, and citrulline. Cbt treatment had a pronounced effect on brain arginine metabolites in PRS rats by significantly increasing homoarginine and reducing homocitrulline to the same levels observed in vehicle-treated unstressed rats.

### 2.5. Enrichment Analysis of Functional Pathways in Plasma and Brain

**Figure 9:**
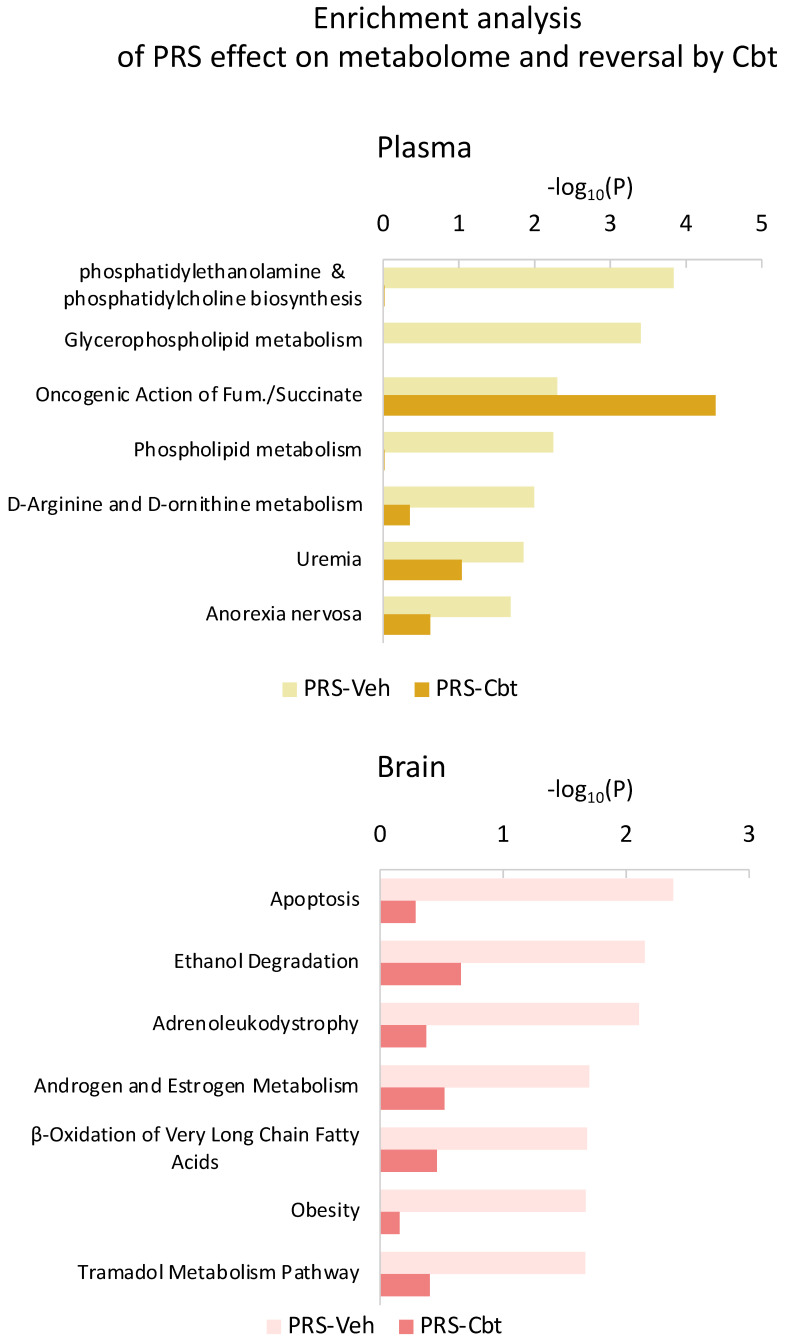
Enrichment analysis in plasma and brain metabolome. Top-ranking HMDB ontology categories enriched for metabolites altered by PRS in the plasma and brain prefrontal cortex, as well as their reversal by Cbt.

We conducted an enrichment analysis to identify functional classes in both plasma and brain metabolomes (Figure 9). In plasma, we observed overexpression of pathways related to phosphatidylethanolamine and phosphatidylcholine biosynthesis, glycerophospholipid metabolism, arginine, and ornithine metabolism, as well as uremia and anorexia nervosa pathways in response to PRS. Remarkably, Cbt retreatment fully corrected the overexpression of these pathways. As for the brain, PRS led to alterations in pathways associated with apoptosis, ethanol degradation, adrenoleukodystrophy, sex hormone metabolism, fatty acids obesity, and antalgic pathways such as tramadol metabolism. Once again, Cbt corrected these alterations.

## 3. Discussion

The objective of this study was to identify metabolomic signatures associated with stress-related disorders during aging and explore the potential reversal of these signatures through oxytocinergic enhancement of maternal care. Additionally, this study investigated hormonal and behavioral correlates that were long-life impaired by perinatal stress and corrected by postpartum carbetocin. This is in agreement with our previous study, except for the cognitive dysfunctions observed in aged PRS rats [12]. Indeed, we observed that aged PRS rats exhibited impaired spatial learning, recognition memory, and social interaction. Postpartum carbetocin corrected these behavioral alterations and the related hormonal dysfunctions by mitigating the reduced maternal care induced by restraint stress during pregnancy. Furthermore, we observed that maternal care is predictive of the behavioral and hormonal profile in aged rats.

Our findings represent the first evidence of how early life stress may alter the metabolomics profile in aged individuals, thereby increasing vulnerability to CNS disorders, and raise the intriguing possibility that pharmacological activation of the oxytocin system soon after delivery through the mother may rectify these alterations by boosting early maternal care. More specifically, we have found that early life stress and oxytocin receptor activation by maternal Cbt cause changes in biochemical markers of distinct metabolic pathways in both plasma and brain. Notably, PRS induced long-lasting changes in biomarkers of secondary bile acid metabolism in the plasma and biomarkers of glutathione metabolism in brain samples. Additionally, we identified shared metabolic changes in both plasma and brain samples related to phenylalanine, tryptophan, benzoate, histidine, and arginine metabolism. Maternal Cbt treatment demonstrated disease-dependent effects by reversing the metabolite alterations induced by early life stress. Furthermore, the metabolomic signatures of early life stress are associated with long-term cognitive and emotional alterations, alongside metabolic and endocrinological disturbances in stress/antistress balance.

PRS resulted in elevated bile acid levels during aging; a phenomenon normalized by carbetocin treatment. Specifically, primary bile acids and precursors were heightened in vehicle-treated PRS rats compared to controls, suggesting altered bile acid synthesis rather than secretion/reabsorption. Bile acids (BAs) play a vital role in lipid emulsification and absorption; they are synthesized from cholesterol via the intermediate 7-alpha-hydroxy-3-oxo-4-cholestenoate (7-Hoca) in the liver and released into the small intestine [25]. Most bile acids undergo enterohepatic circulation and microbial biotransformation in the gut [26]. Most primary BAs are reabsorbed in the terminal ileum and transported back to the liver, while a subset of primary BAs is metabolized by the gut microbiota into secondary BAs, many of which are also reabsorbed. Recent studies have highlighted the role of BAs in the gut–brain axis [27], as the gut microbiota is actively involved in BA metabolism, and BAs may interact with specific receptors present in neurons and influence brain function in physiology and pathology [28]. This suggests a potential pathway through which early life stress-induced alterations in BAs may influence brain function and behavior. Of note, a specific increase in secondary BAs has been reported in other chronic stress models [29]. Cbt reversed changes in BAs induced by PRS, including the PRS-induced increase in muricholate, a major secondary BA in rodents. Interestingly, alterations in BA metabolism have been associated with cognitive impairment in both Alzheimer’s and Parkinson’s disease [30,31,32,33,34,35].

We hypothesize that early life stress augments susceptibility to cognitive dysfunction in later life by modifying BA metabolism, alongside other mechanisms that were mitigated by maternal carbetocin. Metabolites originating from the gut microbiota, such as phenylalanine, tryptophan, and benzoate metabolites were also affected by PRS and Cbt. These and other metabolites generated by the gut microbiota have an established role in the gut–brain axis and may be considered potential biomarkers for health and longevity during aging [36]. Imbalances in the gut microbiota and disruptions in metabolite production may contribute to age-related conditions, impacting an individual’s ability to age healthily.

We also found changes in histidine and arginine metabolism induced by PRS and/or Cbt. Plasma levels of histidine, a precursor of various bioactive compounds like histamine and 3-methylhistidine, were reduced by PRS and restored by Cbt treatment. Among the various histidine metabolites, PRS enhanced both plasma and brain levels of imidazole propionate, which is specifically generated by the gut microbiota. Increased levels of imidazole propionate are produced by subjects with low bacterial gene richness and are also associated with type-2 diabetes mellitus [37], which, in turn, is an established risk factor for CNS disorders, including depression and Alzheimer’s disease. Thus, the enhancing effect of PRS on imidazole propionate levels strengthens the hypothesis that early life stress enhances the vulnerability to CNS disorders later in life and provides further evidence that the gut–brain axis contributes to the neurological and psychiatric outcome of early life stress. Again, an early maternal activation of oxytocin receptors may provide a new strategy to limit the consequences of early life stress. While plasma histamine levels did not differ among our experimental groups, several histamine catabolic products were significantly different by PRS, which may suggest a stimulated histamine turnover. Abnormalities in histaminergic neurotransmission have been reported in several animal models of CNS disorders and are associated with Tourette’s Syndrome and narcolepsy [38,39,40]. Changes in histamine turnover rate, which are suggested by our data on histamine catabolites, provide an additional putative link between early life stress and vulnerability to CNS disorders. PRS also reduced plasma and brain levels of homoarginine and increased levels of homocitrulline. Homoarginine, synthesized ornithine trans-carbamoylase (OTC) or arginine:glycine amidinotransferase (AGAT), is a biomarker of cardiovascular health [41]. In addition, there is evidence of neurotoxicity resulting from homoarginine deficiency [42]. Moreover, PRS induced significant elevation of several plasma urea cycle metabolites, including ornithine and citrulline, suggesting a dysfunctional urea cycle programmed by early life stress. This dysfunction can lead to toxic ammonia accumulation, contributing to neurological dysfunction [43,44,45]. Early Cbt treatment was also able to correct these abnormalities.

The aged brains of PRS rats showed substantial changes in the metabolism of glutathione, which plays a key role in antioxidant defense in neurons and other cell types. Glutathione is a tripeptide composed of glutamate, cysteine, and glycine, and it exists in both reduced (GSH) and oxidized (GSSG) forms. Cysteine and γ-glutamylcysteine synthase (GCS) are the rate-limiting substrate and enzyme for glutathione synthesis, respectively. Cbt increased cysteine levels in the brain of PRS rats without affecting the levels of the cysteine precursor, cystathionine. The cysteine increased in response to maternal Cbt may originate from other precursors, such as methionine or cystine, which is taken up by brain cells through the glycine/glutamate antiporter. The dysregulation of glutathione metabolism caused by PRS and the rescue induced by maternal Cbt treatment are relevant from a translational standpoint because oxidative stress is common to most neurodegenerative disorders, and it also contributes to the pathogenesis of autism and other neurodevelopmental disorders [46]. Of note, it is essential to highlight that evidence suggests a connection between the epigenome and metabolome [47]. The PRS rat model serves as an epigenetic animal model [48] wherein epigenetic mechanisms are partially induced by reduced maternal behavior [12]. Therefore, it is plausible to speculate that metabolomic changes induced by early life stress may also be associated with alterations in the epigenome.

In conclusion, our findings have disclosed novel components of the abnormal developmental programming induced by early life stress, which likely involves the interplay between gut microbiota and brain function. The evidence that Cbt rescued nearly all metabolic alterations caused by PRS by presumably enhancing maternal care in stressed dams lends credit to the hypothesis that mother–child relationships soon after birth are critical for the developmental trajectory of the CNS and also for the metabolic phenotype of the offspring. The evidence that changes caused by early life stress persist during aging strongly suggests the involvement of epigenetic mechanisms. Avoiding chronic stress during pregnancy, and/or improving maternal care soon after birth, may be a valuable strategy to normalize stress response in the adult and aged life, and it may increase resilience to genetic or environmental hits that otherwise cause CNS disorders. An obvious question arises: which mothers should be treated with oxytocin to improve maternal health and alleviate postpartum blues? Brexanolone, a novel FDA-approved treatment for moderate to severe postpartum depression, has shown success. Perhaps measurements of oxytocin levels in the mother after delivery and/or the evaluation of postpartum blues—i.e., the transient and self-limited mild depressive symptoms that may reduce maternal care soon after delivery—may serve as valuable indicators for the selection of mothers who can be treated with oxytocin/carbetocin to prevent abnormal developmental programming of the child. 

## 4. Materials and Methods

### 4.1. Animals

Nulliparous female Sprague-Dawley rats (250–260 g, Charles River, Les Oncins, France) were individually housed with a sexually experienced male for mating. A vaginal smear was performed in order to determine the beginning of the gestation. Presence of spermatozoa defined day 0 of gestation. The animal study protocol was approved by the Comité d’Ethique CEEA-75 (Comité d’Ethique en Expérimentation Animale Nord-Pas de Calais). This project was approved by the MESRI (Ministère de l’Enseignement supérieur, de la Recherche et de l’Innovation) under the APAFIS number #33654.

### 4.2. Perinatal Stress Procedure and Maternal Behavior

Stressed dams were subjected to repeated episodes of restraint stress in a transparent cylinder (7.5 cm diameter, 19 cm long) under a bright light for 45 min three times daily from day 11 of pregnancy until delivery [8]. Control dams were left undisturbed throughout gestation. Maternal behavior was monitored and analyzed as previously described [12]. Control and stressed mothers were placed in standard transparent cages on a rack equipped with cameras. The video recording system included 28 small infrared cameras (CMTH with 1/4 Sony CCD, objective of 3.6 mm) attached to a metal structure and placed about 12 cm distance from the cage wall allowing the whole floor area detection (1 video camera per cage). A constant recording (24 h/24 h) was performed by two infrared LEDs pointed towards the ceiling to provide diffuse IR illumination in the room. Video signals were acquired on two 16 channels DVR encoding H.264 format (Avtech, AVC798ZA, Lille, France). The digital video signal was sent by IP to a computer for storage on a hard disk. Video Viewer Application^®^ (version 0.1.8.4) drove the video recording and replay. From day 1 to day 3 after parturition, for the 2 h following carbetocin or saline injection, the behavior of each mother was scored offline (Noldus, The Observer, Wageningen, The Netherlands) every min for the following active maternal behaviors: arched back nursing, licking, carrying pups. The data obtained correspond to the active presence of the mother on the nest expressed in percent respect to the total number of observations (60 observations/h with 2 h of observation per 3 days, i.e., 121 observations/mother/day).

### 4.3. Carbetocin Treatment

Carbetocin (1 mg/kg, SP080756, Polypeptide group, Strasbourg, France) or vehicle (saline) was administered i.p. to lactating dams from postnatal day (PND) 1 to PND7. The dose and route of administration of carbetocin were selected on the basis of previous reports [12].

### 4.4. Experimental Design

As illustrated in Figure 1, male offspring aged 1–22 months, born to either stressed or unstressed control mothers, which were administered either vehicle or carbetocin were used. The resulting offspring were assigned to four experimental groups for the study: PRS-Veh, PRS-Cbt, and their respective control counterparts (i.e., unstressed and vehicle-treated). Behavioral assessments were conducted between 9:00 a.m. and 1:00 p.m. Brain tissue samples were collected at 22 months of age.

### 4.5. Behavioral Analysis

Spatial recognition: 17-month-old control and PRS rats whose mothers were treated with vehicle or carbetocin were tested using a 2-trial memory task in a Y-maze as previously described [14,49]. The Y-maze (Imetronic, Pessac, France) consisted of 3 identical arms illuminated by a dim light (35 lux) and enclosed by 36 cm high side walls. Each arm was equipped with infrared beams, and the Y-maze was linked to a computer. Numerous visual cues were placed on the wall of the testing room and were kept constant throughout behavioral testing. The floor of the maze was covered with dirty sawdust from the home cages of several animals and was mixed between each passing to eliminate olfactory cues. The task consisted of 2 trials separated by a 6 or 24 h inter-trial interval (ITI). During the first trial (acquisition phase), 1 arm of the Y-maze was closed and animals were placed in the center and allowed to explore the two other arms for 10 min. During the ITI (6 or 24 h), rats were housed in their home cages in a room different from the test room. During the second trial (test phase), the animals had free access to all 3 arms. The parameter evaluated was the time spent in the novel arm (the one closed during the first trial) during the first 3 min of the test phase. This parameter was expressed in percentages and was compared with the percentage of random chance exploration of the 3 arms (i.e., 33% for each arm). The animals were determined to have discriminated between the novel arm and the 2 familiar arms if the percentage of time spent in this arm was significantly superior to 33%. Memory performance was tested using 2 ITI (6 and 24 h) separated by an interval of 1 week.

Social interaction: Control or PRS rats whose mothers were treated with vehicle or carbetocin were tested at 18 months old. The juvenile recognition ability was assessed using a procedure adapted from [50]. Rats were individually placed in transparent cages for 5 min for habituation. The challenged rat was first exposed to a male juvenile (2-month-old) for 5 min. Rats were exposed to a different juvenile 24 h and one week later (three different juveniles were used to avoid habituation between sessions). Sessions were video-recorded, and the time spent in interaction (sniffing, grooming, anogenital, and play behavior) was measured by a trained observer using the Observer 20 (Noldus, Wageningen, The Netherlands).

Open field: Control and PRS rats whose mothers were treated with vehicle or carbetocin were tested at 19 months old. Exploratory behavior [15] was evaluated by placing a rat into a corner of an open-field arena (100 cm × 100 cm × 50 cm), allowing the rat to explore the field for 10 min freely. Lightning was approximately 60 lx inside the arena.

Activity and trajectory length in the open field were recorded and quantified by Video Track^®^ (Viewpoint, Lyon, France). The time spent in the periphery, corners, and center of the apparatus were recorded.

Spatial learning: 20-month-old control and PRS rats whose mothers were treated with vehicle or carbetocin were assessed in the Morris water maze test [51]. A plastic tank (2 m in diameter, 0.6 m in height) was filled with water (22 ± 2 °C) up to 35 cm. Spatial cues were placed in the room and remained fixed throughout the experiment. Before the test, animals were submitted to a two-day habituation phase, during which they were left for 1 min to explore the pool. During the test, animals were required to find a hidden platform (20 cm diameter) placed 3 cm below the water surface. The walls of the tank and the platform were black and indirect lightning was used in the room, enabling the platform to be hidden from the animals’ sight. All rats were submitted to 3 trials per day for 6 days, and the starting positions changed over the trials. Each trial began with the animal in the pool facing walls and ended either after 90 s of swimming or when the animal found the platform. In either case, the rat was left on the platform for 20 s. Latency and distance to reach the platform were recorded using an automated system (Viewpoint, Lyon, France).

### 4.6. Physiological Analysis

Body weight gain: The body weights of the progeny were assessed at 17, 20, and 22 months of age. Data were expressed as absolute weight (g).

Peripheral markers: Glucose (mg/dL), insulin (ng/mL), corticosterone (ng/mL), and oxytocin (pg/mL) measurements were performed in aged control and PRS progeny of dams treated with either saline or carbetocin during the postpartum period. To assess blood glucose regulation, we conducted measurements of glucose and insulin secretion after carrying out the study of spatial recognition memory in the Y-maze test. Glycaemia was measured with a glucometer (Accu-check^®^ Aviva; Roche Diagnostics, Indianapolis, IN, USA), following a small incision of the tail vein. Insulin measurement was performed in plasma obtained from blood samples collected from the tail vein as well. After the behavioral testing in the Morris water maze, blood was collected for measurements of corticosterone and oxytocin levels. Blood (approximately 200 μL each) was carefully collected in tubes containing EDTA (2 mg/mL). Following centrifugation at 1500× *g* for 15 min at 4 °C, the plasma was separated and subsequently frozen at −20 °C until further analysis. Insulin, corticosterone, and oxytocin were quantified using an enzyme immunoassay mentioned in Table 1. All enzyme-linked immunosorbent assay (ELISA) kits were used according to the manufacturer’s protocol. All standards, blood samples, and controls were analyzed concurrently in duplicate. The optical density of the samples was determined at 450 nm using a microplate reader (BioTek Instruments, Winooski, VT, USA).

### 4.7. Metabolome Analysis

Plasma and brain metabolic profiles were measured using the untargeted metabolomics platform from Metabolon Inc. (Morrisville, NC, USA). The plasma and frontal cortex were collected at the end of the experimental design and maintained at −80 °C. Samples were prepared using the automated MicroLab STAR^®^ system from Hamilton Company (Reno, NV, USA). Several recovery standards were added before the first step in the extraction process for QC purposes. To remove protein, dissociate small molecules bound to protein or trapped in the precipitated protein matrix, and recover chemically diverse metabolites, proteins were precipitated with methanol under vigorous shaking for 2 min (Glen Mills GenoGrinder 2000, Clifton, NJ, USA) followed by centrifugation. The resulting extract was divided into five fractions: two for analysis by two separate reverse phases (RP)/UPLC-MS/MS methods with positive ion mode electrospray ionization (ESI), one for analysis by RP/UPLC-MS/MS with negative ion mode ESI, one for analysis by HILIC/UPLC-MS/MS with negative ion mode ESI, and one sample was reserved for backup. Samples were placed briefly on a TurboVap^®^ (Zymark, Hopkinton, MA, USA) to remove the organic solvent. The sample extracts were stored overnight under nitrogen before preparation for QA/QC analysis. Several types of controls were analyzed in concert with the experimental samples: a pooled matrix sample generated by taking a small volume of each experimental sample served as a technical replicate throughout the dataset; extracted water samples served as process blanks; and a cocktail of QC standards carefully chosen not to interfere with the measurement of endogenous compounds were spiked into every analyzed sample, allowing instrument performance monitoring and aiding chromatographic alignment. Instrument variability was determined by calculating the median relative standard deviation (RSD) for the standards that were added to each sample before the injection into the mass spectrometers. Overall process variability was determined by calculating the median RSD for all endogenous metabolites (i.e., non-instrument standards) present in 100% of the pooled matrix samples. Experimental samples were randomized across the platform run with QC samples spaced evenly among the injections.

### 4.8. Statistical Analysis

Behavioral and physiological data were analyzed by two-way ANOVA or ANOVA for repeated measures as indicated. The Neuman–Keuls post hoc test was used to isolate the differences. Correlations were analyzed using Pearson’s correlation analysis. Significance was set to *p*-value < 0.05.

In the metabolomics analysis, following log transformation and imputation of missing values, if any, with the minimum observed value for each compound, a two-way ANOVA was used to identify biochemicals that differed significantly between experimental groups. The false discovery rates were calculated on a per-test basis using Story’s q-value. For differential metabolite concentration analysis in the Volcano plots, Welch’s *t*-tests were used. False discovery rates were calculated by Benjamini–Hochberg. Outliers were first determined by the “outer fence” criteria (3rd quartile + IQR) and were replaced by the next highest value. The significance of Euclidian distances between groups was calculated by PERMANOVA. For pathway enrichment analysis, the labeling of metabolites into metabolite sets was taken from the Human Metabolome Database (HMDB). We used a “mean rank” test, testing whether the mean rank of metabolites in a set significantly differs from what is expected by random. To do this, all measured metabolites were ranked by their group-comparison *p*-values, and the ranks were then normalized to the range (0, 1). Under the null hypothesis, the mean of the ranks can be approximated as 0.5 with standard deviation sqrt (1/12 n); the true mean rank was compared with the null with a z-test. Data were expressed as −log10(P).

## Figures and Tables

**Table 1:** Description of used ELISA kits.

ELISA Kit	Manufacture	Sensitivity	Intra CV (%)	Inter CV (%)
Insulin-rat	Demeditec (DEV 8811) Kiel, Germany	0.1 ng/mL	<6%	<9.5%
Corticosterone rat/mouse	Demeditec (DEV 9922), Kiel, Germany	6.1 ng/mL	<9%	<8.5
Oxytocin rat	CUSABIO (CSB-E14197r), Houston, TX, USA	9.4 pg/mL	<15%	<15%

## Data Availability

Data is contained within the article and Supplementary Material.

## Supplementary Materials

The following supporting information can be downloaded at: https://www.mdpi.com/article/10.3390/ijms25053014/s1.
