# Supplementary material for: Postpartum Oxytocin Treatment via the Mother Reprograms Long-Term Behavioral Disorders Induced by Early Life Stress on the Plasma and Brain Metabolome in the Rat
Source: Int J Mol Sci. 2024 Mar 5;25(5):3014. doi: 10.3390/ijms25053014 (PMC10932010; doi:10.3390/ijms25053014)
Supplement: Supplementary file 1 [file ijms-25-03014-s001.zip › ijms-2877200-supplementary.pdf]

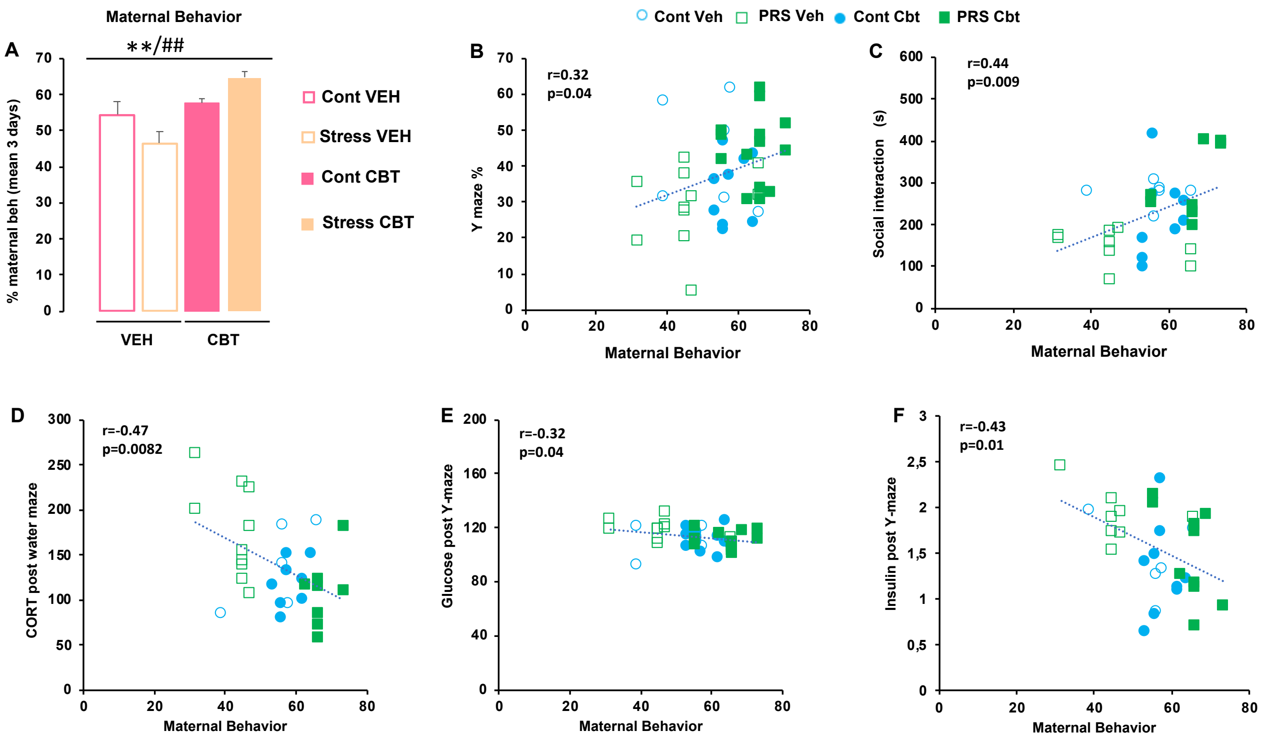


Supplementary Figure S1. Programming of PRS phenotype by maternal behavior. A) Maternal care (nursing/licking behavior) during the first 3 postpartum days i(n=8-15 dams/group). Values are expressed as means ± S.E.M. * indicates PRS effect and # indicates Cbt effect. Specifically, in the figure: PRS x Cbt interaction **/## p<0.01; Correlation analysis of offspring behavioral (B-C) and hormonal (Figures D-F) parameters with maternal care (nursing/licking behavior during the first 3 postpartum days. Pearson's correlation coefficient (r) values and related p values are reported.
